# Toward Family-Centered Patient Portals for Childhood Asthma Management Among Black Primary Caregivers: Qualitative Interview Study

**DOI:** 10.2196/100502

**Published:** 2026-09-24

**Authors:** Aviv Y Landau, Anthonia Odarkor Odartei, Levia Airall Sutton, Leila Azari, Jamie Flores, Hita Kambhamettu, Sonya Sanders, Andrew Head, Kenrick Cato

**Affiliations:** 1 School of Social Policy and Practice, University of Pennsylvania, 3901 Walnut, Philadelphia, PA, United States, (215) 746-6290; 2 School of Nursing, University of Pennsylvania, , Philadelphia, Pennsylvania, United States; 3 USC Dworak-Peck School of Social Work, University of Southern California, Los Angeles, CA, United States; 4 Perelman School of Medicine, University of Pennsylvania, Philadelphia,, Philadelphia, United States; 5 Computer and Information Science, University of Pennsylvania, Philadelphia, PA, United States; 6 Philly Thrive, Philadelphia, PA, United States; 7 University of Pennsylvania School of Nursing, Children's Hospital of Philadelphia, University of Philadelphia, Philadelphia, PA, United States

**Keywords:** childhood asthma, electronic health records, health portals, caregivers, qualitative research, health equity, community-based research

## Abstract

**Background:**

Childhood asthma places substantial burdens on caregivers, particularly in communities facing structural barriers to health care access. Patient portals, the patient-facing component of electronic health record systems, are primarily designed for clinical workflows, limiting their usefulness for caregivers managing their child’s asthma.

**Objective:**

This qualitative study aimed to elicit primary caregivers’ perspectives on managing childhood asthma and their experiences with patient portals to inform the development of a community-centered and family-centered medical platform.

**Methods:**

The authors conducted a qualitative study using in-depth, semistructured interviews between July and October 2024 with 15 Black primary caregivers of children with asthma living in the Greater Philadelphia area. Participants were recruited through purposive sampling in partnership with Philly Thrive, a community-based organization. Interviews were conducted both in person and virtually and lasted 30 to 90 minutes. A semistructured guide with 3 core questions explored experiences with managing childhood asthma, interactions with medical documentation and patient portals, and recommendations for redesigning patient portals. The number of interviews was determined by data richness and fulfillment of study aims. Data were analyzed using reflexive thematic analysis, guided by the Standards for Reporting Qualitative Research.

**Results:**

Two themes were generated. Theme 1, lived experiences of managing childhood asthma in families and communities, revealed that caregiving involves profound emotional work, daily routines, and community knowledge sharing that health systems largely do not recognize or support. Theme 2, caregivers’ perceptions and experiences of navigating patient portals for childhood asthma, revealed that caregivers used portals selectively at key care moments rather than routinely. Persistent barriers included confusing medical terminology, outdated medication records, and limited communication channels. Caregivers recommended direct provider messaging, integration of environmental trigger data, streamlined medication refill processes, and age-appropriate portal features to support children’s gradual self-management.

**Conclusions:**

Effective childhood asthma management requires information systems that honor caregiver expertise, provide transparent and accurate documentation, and actively work to reduce epistemic injustice. Community-centered platform design should integrate caregiver knowledge, environmental data, and child-appropriate tools to support equitable asthma care.

## Introduction

### Background

Childhood asthma affects approximately 4.9 million children in the United States and is one of the most common chronic conditions in childhood, as well as a leading cause of missed school days and health care usage [1]. Globally, the condition affects approximately 10% of children and is characterized by symptoms such as wheezing, coughing, and shortness of breath that disrupt daily activities [2]. While asthma cannot be cured, it can be managed through ongoing treatment that requires consistent daily care, both inside and outside clinical settings.

The majority of childhood asthma management occurs outside clinical settings. Primary caregivers take responsibility for monitoring symptoms, administering medication, and coordinating care [3]. Recurrent exacerbations, emergency department visits, and disruptions in school attendance place emotional and practical burdens on families [4]. These challenges are frequently more pronounced in Black and Hispanic communities, which bear a disproportionate burden of asthma morbidity rooted in structural racism, including residential segregation, exposure to poor housing conditions, and disproportionate proximity to environmental pollutants that worsen air quality and increase asthma risk [5,6]. Pediatric asthma is defined as a chronic inflammatory condition of the airways characterized by variable airflow limitation and respiratory symptoms, including wheezing, coughing, and shortness of breath [7]. Current management encompasses a stepwise pharmacological approach, from inhaled corticosteroids to biological therapies, including omalizumab and dupilumab, for children with severe or poorly controlled disease [8,9].

Although effective treatments exist, many children continue to experience poorly managed symptoms in real-world settings, contributing to ongoing disease burden and health care usage [10]. Effective management depends on more than treatment availability. Collaboration between caregivers and providers, including clear communication about treatment plans, is essential for ensuring families can manage symptoms and medications at home [11].

The electronic health record (EHR), an evolving technology with widespread use, supports communication and shared decision-making between families and providers; however, limited caregiver engagement signals persistent barriers to its use [12]. EHR systems are primarily designed for clinical practice rather than patient or caregiver involvement. This design gap limits accessibility and has been described as a form of epistemic injustice: a harm done to someone specifically in their capacity as a knower [13,14]. These gaps suggest that EHR systems, designed primarily for clinical workflows, may be misaligned with the realities of how caregivers implement and navigate asthma management. Interdisciplinary and community collaboration offers a path toward addressing these barriers by reimagining how medical documentation is designed to better integrate community knowledge to meet caregiver needs [15,16].

These design gaps are compounded by health literacy challenges: caregivers who already struggle to understand medical information face additional barriers when EHR interfaces require them to navigate complex clinical terminology without support [17,18]. Variability in how clinical information is documented across systems further limits caregivers’ ability to build a coherent picture of their child’s health [19]. These inequities extend into the digital health environment, where patient portals designed primarily for clinical workflows may further disadvantage Black caregivers by failing to support the realities of their caregiving context [20,21]. This study, therefore, centers the perspectives of Black primary caregivers as those most affected by these intersecting inequities and least served by current portal designs.

Despite growing recognition of these disparities, qualitative research examining how Black primary caregivers specifically experience and navigate patient portals in the context of managing childhood asthma remains limited [12,20,22,23]. This study addresses that gap by centering caregivers as domain experts whose lived experiences are essential to designing health information systems that work for the communities most burdened by asthma.

### Objective

This qualitative study aimed to elicit primary caregivers’ perspectives on managing childhood asthma and their experiences with patient portals (the patient and caregiver-facing system of an EHR) in their child’s care and to inform the development and implementation of a community-centered and family-centered medical platform.

## Methods

### Study Design

We conducted a qualitative study using in-depth, semistructured interviews with 15 caregivers of children with asthma, focusing on their experiences with patient-facing portal interfaces rather than clinician-facing EHR systems, as these tools present distinct design challenges for caregiver engagement. The aim was to generate community-informed insights into caregivers’ information needs and their interactions with clinical documentation systems.

### Participants

All participants were Black female caregivers of a child with asthma living in the Greater Philadelphia area. For this study, a primary caregiver was defined as an adult who identified as the person primarily responsible for the day-to-day health management of a child with an asthma diagnosis. Recruitment was conducted in partnership with Philly Thrive, a community-based organization serving the region. This partnership considered caregivers as domain experts whose lived experience was central to understanding asthma management, health information needs, and the limitations of current patient portal systems. We focused on Black primary caregivers because Black children experience disproportionately high asthma morbidity in the United States, and Black caregivers often manage asthma within conditions shaped by structural racism, environmental inequity, and unequal health system responsiveness [5,6]. Centering this population allowed the study to examine patient portal design and EHR documentation in asthma management from the perspective of caregivers most affected by these intersecting inequities. Asthma diagnoses were caregiver-reported and ranged from intermittent to persistent; the study did not collect formal spirometry or medical record data on asthma classification.

The sample consisted of 15 caregivers with an average age of 41.7 (SD 7.28, range 27‐55) years, each managing the treatment of at least one child with an asthma diagnosis. The participants included single primary caregivers (n=13) and married primary caregivers (n=2). Primary caregivers had between 1 and 8 children (mean 3.87, SD 2.03). All participants reported English as their primary language and accessed care for their child with asthma through various pediatric health systems in the Greater Philadelphia.

### Procedure

In-depth one-on-one interviews were conducted between July 2024 and October 2024. The number of interviews was determined by data richness and by the fulfillment of study aims, which were assessed through ongoing review of transcripts [24]. AOO, a social work student trained in qualitative research methods with experience in health equity, conducted all interviews. All interviews were audio-recorded and video-recorded and transcribed verbatim. Primary caregivers received a US $100 gift card in recognition of their time and contributions to the research. We recognize that participants from marginalized communities contribute not only their time but also their expertise and the emotional labor of sharing lived experiences of health inequity; future community-based research should consider compensation structures that more fully reflect this contribution.

A semistructured interview guide was used to explore participants’ experiences with clinicians and with medical documentation regarding their child’s asthma. The guide included the following questions: (1) What was your first conversation with your clinician about your child’s asthma? (2) Tell us about instances you can remember learning about your child’s asthma through medical documentation and what you understood from these documents. (3) What would you do if you had the opportunity to redesign medical documentation concerning your child’s asthma? Interviews were conducted in person and via Zoom and lasted 30 to 90 minutes. Quotations presented in the “Results” section were lightly edited for readability (eg, removal of false starts and filler words) while preserving the speaker’s meaning and voice.

### Ethical Considerations

The study received approval from the University of Pennsylvania Institutional Review Board prior to data collection (protocol number: 23‐1810). Participants provided informed consent before participation, and all transcripts were deidentified to protect confidentiality. To further protect participant privacy, all names reported in this paper are pseudonyms assigned by the research team; no real names are used. Participants were informed of the sensitive nature of the research topic and their right to withdraw at any time.

### Data Analysis

Reflexive thematic analysis was conducted in 3 stages to generate key themes and categories [25,26]. This study followed the SRQR (Standards for Reporting Qualitative Research) reporting guideline [27] (Checklist 1). Drawing on Braun and Clarke’s framework for quality in reflexive thematic analysis, we prioritized reflexivity, rigor, and thick description as ongoing analytic practices rather than criteria to satisfy [25,26]. Reflexivity was central to the process: AYL is a social worker and health informatics researcher whose prior work on EHR design and marginalized communities shaped the study questions, theoretical framing, and interpretive lens. AOO is a social worker with experience working with marginalized communities facing health disparities, and LAS is a nurse and doctoral student with clinical experience caring for people living with chronic conditions across the lifespan. Both researchers maintained reflexive discussions throughout data collection and analysis to examine how their subjectivities and assumptions actively shaped the interpretive process. Thick description of the study context, participant characteristics, and analytic process is provided throughout to support readers in assessing the relevance of findings to other settings. In stage 1, AYL and AOO engaged in deep familiarization with the data, repeatedly reading all interview transcripts as an active interpretive process rather than a neutral scan of content. Consistent with Braun and Clarke’s reflexive approach, this stage foregrounded the researchers’ subjectivities as resources rather than sources of bias, acknowledging that engagement with data is always shaped by the researcher’s perspective [25,26]. Using Dedoose (SocioCultural Research Consultants, LLC), a qualitative software platform, both researchers independently coded 2 identical transcripts, attending to meaning and interpretive possibilities rather than surface content. Codes were compared and discussed within the research team to surface interpretive differences, refine the coding approach, and develop a working code set. In this phase, we constructed codes such as “Lived experience with managing asthma” and “Perceptions about patient portals.”

In stage 2, the remaining transcripts were coded with active reflexive engagement, and codes were continuously interrogated and revised in relation to the dataset as a whole. Rather than mechanically sorting codes into predetermined groups, AYL and LAS worked to identify patterns of shared meaning across the data, constructing candidate themes that represented coherent interpretive accounts of caregivers’ experiences. Throughout this nonlinear process, codes and themes were added, collapsed, or reconsidered as interpretations developed. LAS, a doctoral student in nursing, joined the analytic team in stage 2 after AOO completed data collection responsibilities. LAS reviewed the stage 1 codebook in full, discussed the rationale for each code with AYL, and conducted independent verification coding on 2 transcripts before proceeding to stage 2 analysis. Analytic decisions and interpretive choices were documented and discussed as a team throughout. For example, the initial code “Routine usage of patient portal” was examined to understand whether and how caregivers used patient portals. This analysis led to 2 new codes: “Positive experiences with patient portal” and “Negative experiences with patient portal,” which were grouped into the category “Accessing health records for care needs.”

In stage 3, the research and community team collaboratively reviewed and defined each theme, ensuring that it told a coherent and grounded story in relation to the dataset as a whole. Theme names were developed as interpretive acts, chosen to be vivid and analytically meaningful rather than simply being descriptive. For example, the candidate themes “The emotional and practical work of asthma care” and “Building understanding to support asthma management” were brought together and defined as the central theme “Lived experiences of managing childhood asthma in families and communities,” capturing the relational and community-embedded nature of caregiving that ran across both themes.

## Results

### Overview

Two themes were generated from the analysis: (1) lived experiences of managing childhood asthma in families and communities, encompassing the emotional, practical, and social dimensions of asthma caregiving; and (2) caregivers’ perceptions and experiences of navigating patient portals for childhood asthma, capturing how caregivers access patient portals and medical records, the barriers they encounter, and their recommendations for improvement.

### Theme 1: Lived Experiences of Managing Childhood Asthma in Families and Communities

This theme highlights how managing a child’s asthma is an emotional, relational, and practical process embedded within family life and community (Table 1). A distinct type of fear runs through many caregivers’ accounts, arising from watching their child struggle to breathe, intensified by the reality that children may not fully understand or communicate the seriousness of their symptoms (Q1-Q2). This emotional burden is inseparable from the practical, everyday work of asthma care. Toni’s account—constantly reminding her son to take his medications, laying them out each morning before she leaves for work, and rehearsing inhaler technique with him the night before—makes this interplay of vigilance and routine concrete (Q3).

**Table 1.** Theme 1: Lived experiences of managing childhood asthma in families and communities.

Subthemes	Illustrative quotes
Emotional and practical work of asthma care	Q1: *As a parent, to see your children struggle, oh my god. That’s a different type of fear*. [Natasha] Q2: *The advice that I would like to give is to be mindful, always be alert. I think it’s vitally important to be alert, mindful, and watchful, especially when watching a child, because children are so innocent and may not fully understand the seriousness of certain things*. [Laura] Q3: *I constantly speak, talk to him, and let him know it is very important that he takes his medication. Make sure that his medication is laid out on the sink each morning, as I leave home before he leaves for school. And so, in case my daughter, who takes him to school, forgets, I make sure that each night the medication is laid out and remind him in the morning before I leave: “Make sure you shake your pumps up before you take them.” And so basically, I have these talks quite often*. [Toni]
Building understanding to support asthma management	Q4: *They (health workers) talked to me. They broke it down and explained exactly what asthma is, what can cause flare-ups, how to treat it, and how to manage it*. [Maya] Q5: *I had asthma classes. Asthma studies. I think that’s really good ’cause you learn more and more about your child’s health, which can help decrease the number of times you have to go to the hospital*. [Katherine] Q6: *If you’re going into the hospital, I would think maybe flyers would be more effective because your appointment is only for a specified amount of time. However, if you’re going into schools, webinars will most certainly be of interest to parents, allowing them to sit, watch, and learn. It might be more interesting than just hearing somebody speak*. [Denise] Q7: *I want the medical field to take asthma a whole lot more seriously. Because you have parents at home, that’s literally just like losing their mind. The child feels like they’re losing their life, and the mom or the dad feels like they’re losing their mind. I never in life had to make this decision, but I did last week, and I was just like you’re going to go home with daddy because I have to go to work, and if you’re home with me, I have to be up all night and still get up for work at 6:00 o’clock. And my baby screamed and hollered and cried and looked me in my eyes and said, you need to tell them your child is sick*. [Natasha]
Confidence through shared experience and support	Q8: *When my 14-year-old had her first asthma attack at the age of three, because I have asthma, I knew the signs and symptoms*. [Keesha] Q9: *It was easier because she knew (grandmother) what to do because both my sister and my dad had it (asthma), so it definitely killed my nervousness or anxiety*. [Gabby] Q10: *So everyone in my support system knows what to do, what to look for. We are basically familiar with this asthma thing. It’s unfortunate, but we’re just on point with it*. [Toni]

Building understanding through clear and accessible information was central to effective asthma management. Caregivers valued plain-language explanations from health workers and participation in asthma education programs, which some believed reduced hospital visits (Q4-Q5). When asked how information could be better delivered, caregivers offered format-specific suggestions based on the demands of different settings: flyers might work better in time-pressured hospital appointments, while webinars could allow parents in school settings to learn at their own pace (Q6). These accounts reflect caregivers’ active pursuit of knowledge and the considerable effort it requires. Natasha’s account makes the weight of that effort concrete: she describes the medical field’s failure to take asthma seriously as something felt in the body, in sleepless nights, and ultimately in an impossible choice between her child’s safety and her livelihood (Q7). Work and caregiving demands do not simply compete; they reveal the structural and economic pressures that make asthma management far harder than any educational program alone can address.

For many participants, personal and family experience with asthma served as a source of confidence and readiness. Those who had asthma themselves or had family members with the condition felt better equipped to recognize early warning signs and respond quickly (Q8-Q9). This shared knowledge extended to broader support networks, with participants ensuring that everyone involved in their child’s care knew what to look for and what to do during an episode (Q10). Such collective familiarity, though born of difficult circumstances, provided reassurance that children would be safe even when caregivers were absent. In this sense, the practical knowledge caregivers have accumulated over years of managing asthma within their families constitutes a form of clinical familiarity that is neither captured in nor recognized by existing documentation systems.

### Theme 2: Caregivers’ Perceptions and Experiences of Navigating Patient Portals for Childhood Asthma

This theme captures how primary caregivers access, interpret, and respond to patient portals and medical documentation, as well as the challenges they encounter in doing so (Table 2). Engaging with the patient portal was a selective, need-based practice, shaped by time constraints, trust in existing systems, and the immediacy of care demands. Rather than consulting records routinely, Denise relied on automated processes such as prescription refills, accessing documentation only when changes arose or when clarification was needed (Q11). Gabby used the portal to verify clinical information following appointments, particularly when co-parenting arrangements limited direct communication about what had been discussed with the doctor (Q12). Katherine described using the portal to send a doctor’s note directly to her child’s school, eliminating the need to print and hand-deliver paper documentation (Q13). Alicia highlighted a specific limitation of current portal systems: records from visits to different hospitals or health systems are not automatically consolidated in one place, and her suggestion that all visits from any provider appear in a single portal reflects the reality that patients who receive care across multiple institutions must navigate fragmented records that do not follow them from provider to provider (Q14).

**Table 2.** Theme 2: Caregivers’ perceptions and experiences of navigating patient portals for childhood asthma.

Subthemes	Illustrative quotes
Accessing health records for care needs	**Q11:** *I’m very busy, I don’t have time to go in and look for stuff. But I’ll refer when I need to refer to anything. But his (child) prescriptions have been with his doctor and his pharmacy forever, so they’re like on automatic refill unless he runs out or the dosage changes. So, there’s really no need for me to keep doing all of that because they do it*. [Denise] **Q12:** *I normally just compare his progress or their progress. I look from last year to this year. Make sure it’s nothing I missed since I don’t take him to the doctor. His dad is not really good at explaining what the doctors say. I work in healthcare, and I can kind of figure some things out. But I just like to go in there to read and make sure the paperwork matches what’s in the portal*. [Gabby] **Q13:** (Caregivers) *Send a note through your medical record to give to your teacher*. [Katherine] **Q14:** *I would suggest they put everything onto the portal. In case you lose the papers, you can still see everything (all medical information across medical institutions and settings). All visits from whatever doctor that patient sees should all be in one place*. [Alicia]
Barriers to understanding and accuracy	**Q15:** *They have to remove it (old prescriptions) because it is still there. When she gets other medicines, they just add up. And I have to go and tell ’em like, you gotta take that off and that off because she doesn’t take that anymore. So I don’t want them to think that that’s what she takes*. [Alicia] **Q16:** *It’s so much. It’s not easy. It’s a lot. You have to figure out what....If it’s an app or a website, I want it to be easy to understand. I don’t have patience for figuring it out when my child is sick*. [Natasha] **Q17:** *I honestly, in my opinion, feel like there are things that the medical professional knows and is knowledgeable of that we don’t....That they don’t share with us, for whatever reason. So, I definitely would appreciate and love to take advantage of an AI’s assistance when it comes to helping me review notes, charts, and gain an understanding of what’s going on*. [Laura] **Q18:** *I had to do too much to get certain diagnoses when I already knew what the problem was. No parent should have to go through that. You had to jump through hurdles to get results*. [Jill]
Caregivers’ recommendations for improving patient portals	**Q19:** *I would also like to reach out to the physicians on the app, instead of waiting for a phone call, or having to constantly call and call*. [Christina] **Q20:** *Every day, I’m looking at the pollen levels for him. Every day, because it’s like, okay, if the pollen levels are high, we cannot go outside*. [Nicole] **Q21:** *So, say if my child was having symptoms that I’m unfamiliar with, I think I should be able to go on the app and search those symptoms, and then I think the app should have a contact where the doctor or nurse, whoever reviews it, is able to get in contact with you. Like we, as the parent, get to leave our information so the professionals can get back to us*. [Toni] **Q22:** *When you are diagnosed with asthma, you should be able to get a refill for your child’s asthma pump as soon as possible. You should not have to set up a doctor’s appointment. They already have the diagnosis, so it should just be sent to the pharmacy*. [Shonte]
Fostering autonomy in children living with asthma	**Q23:** *I told my daughter to download it. You can send messages to your doctor yourself at 13. You’ve got a problem, go to your doctor. I can always see it. But you can go to your doctor on your own. So, he’s turning 18 soon, he’s gonna have access to his own chart, and they better not limit me, but I think they will*. [Gabby] **Q24:** *I actually think the health portal should be easy for anybody. I use it for myself too. I recently had blood work done and was able to look at the results with definitions right there. I believe children could use it the same way*. [Simone] **Q25:** *Now that she’s getting older, I’m starting to tell her what’s what and how it means for her, or how to take care of herself in regard to that. Now, more because I feel like she understands more now*. [Maya]

Many participants described having to exert substantial effort to obtain accurate information, correct errors, and secure recognition of what they already knew about their child’s condition. Alicia described having to actively advocate for the removal of outdated medications from her child’s record concerned that persistent errors could lead to clinical mistakes (Q15). Many participants expressed frustration that critical information was not always fully shared or explained to them (Q16). Laura felt excluded from aspects of her child’s care and expressed interest in AI-assisted tools to help interpret medical notes and bridge the knowledge gap between herself and providers (Q17). Jill described having to fight repeatedly to secure her child’s diagnosis, reflecting a broader pattern in which caregivers must exert significant effort simply to be heard (Q18).

Participants offered concrete recommendations for improving the patient portal, grounded in their daily experiences. Direct messaging with physicians was a consistent priority, with Christina describing frustration with having to wait for phone calls rather than sending a message through the app (Q19). Nicole described checking pollen levels daily to inform decisions about her child’s outdoor activity (Q20). While environmental data are available through weather applications, integrating them directly into the patient portal may allow caregivers to connect real-time trigger information to their child’s documented symptoms and medication use. Toni proposed a symptom-search feature that would allow parents to look up unfamiliar symptoms and receive clinical follow-up (Q21). Shonte highlighted unnecessary friction in medication refills, arguing that a confirmed diagnosis should be sufficient to authorize a prescription without requiring a new appointment (Q22).

Several participants described gradually involving their children in managing their own asthma care. Gabby encouraged her daughter to use the patient portal independently starting at age 13, sending messages to her own doctors while still keeping a parental view of the account (Q23). Simone noted that the health portal should be accessible and easy for anyone to use, including children, pointing to its potential for youth engagement with health information (Q24). As Maya explained, she began sharing more details about asthma management as her daughter grew older and could better understand what it meant for her health (Q25). Taken together, these accounts suggest that caregivers view the patient portal not only as a tool for adult management but also as a scaffold for children’s gradual development of health self-agency—a function that current portal design does not explicitly support.

## Discussion

### Principal Findings

This qualitative study offers several insights from Black primary caregivers about their lived experiences managing childhood asthma and their interactions with EHR-generated information, patient portals, and medical documentation. These perspectives contribute to the development of a community-centered and family-centered medical platform that addresses caregivers’ information needs while reducing epistemic injustice in health care systems [13]. Two primary themes illustrate how caregivers navigate childhood asthma care and engage with health information systems. The first illuminates the lived experience of childhood asthma caregiving, encompassing the emotional weight, daily routines, and community knowledge that sustain management outside clinical settings. The second turns to caregivers’ engagement with patient portals, revealing patterns of selective use, persistent barriers to accurate and accessible information, and a set of grounded recommendations for redesign.

Caregivers described the emotional and practical day-to-day work of asthma care as an ongoing process that extends beyond the provision of medication. They articulated a distinct type of fear associated with watching their child struggle to breathe, intensified by the reality that children may not fully understand or communicate their symptoms. The ongoing work involves establishing daily routines, preparing medications, and providing reminders to ensure treatment adherence. These findings are consistent with prior research documenting caregiver burden associated with childhood asthma [3,4] and underscore opportunities for technology to reduce the informational and logistical burden of daily asthma management, particularly for caregivers navigating care largely outside clinical settings.

Caregivers consistently described clear, accessible information as central to their ability to manage their child’s asthma confidently. They valued plain-language explanations from health workers and participation in asthma education programs, which some believed had reduced hospital visits. These findings support previous research on health literacy and asthma management [17,18] and add specificity regarding preferred educational formats. Caregivers suggested that flyers may be more effective in busy hospital settings, while webinars may be useful in school contexts, as they allow parents to learn at their own pace. These preferences suggest opportunities to integrate multimodal educational content that adapts to different caregiving contexts.

Managing childhood asthma is inseparable from the conditions of daily life. When work obligations and caregiving responsibilities collide, caregivers face choices that no health information system can resolve, yet that directly shape their ability to manage their child’s condition. These tensions are consistent with research documenting the compounding demands faced by caregivers, particularly Black caregivers, who disproportionately face employment precarity and insufficient institutional support when managing chronic illness in their children [5,6]. Platform design should therefore support caregivers not only in understanding medical information but also in navigating the broader competing demands of daily life.

Families’ lived experience with asthma created expertise in managing the condition. Caregivers with a personal or family history of asthma felt better equipped to recognize early warning signs and respond quickly. These findings align with prior research on caregiver knowledge in pediatric chronic illness management and extend this work by demonstrating how family knowledge and lived experience serve as strengths in childhood asthma management [3,4]. Lived experience represents a form of domain expertise that is often unrecognized in health information systems, suggesting opportunities to enhance the sharing of experiential knowledge alongside professional medical knowledge.

Caregivers did not describe patient portals as resources for routine, everyday asthma management. Rather, they used them selectively at times when documentation was needed for coordination, verification, or urgent decision-making, such as reviewing what was discussed at appointments, sharing information with schools, confirming medications, or referencing medication details during emergency care visits. Seen this way, selective use reflects not low engagement but the limited usefulness of current systems for the day-to-day work of asthma care [12,20,21]. This pattern is consistent with evidence from a study of diverse caregivers of children with asthma showing that limited digital health app engagement reflects barriers related to design and accessibility rather than caregiver willingness or motivation [22].

Caregivers described encountering records that were outdated, difficult to understand, and sometimes incomplete. Medication lists retained prescriptions their children no longer took, requiring caregivers to intervene directly to prevent errors. Clinical terminology left caregivers searching elsewhere for explanations, and many felt providers did not share the full picture of their child’s condition. These findings reflect broader patterns of medical mistrust among Black patients, which research has linked to histories of discrimination and perceived asymmetries in clinical encounters [28]. This dynamic extends into digital communication channels: research demonstrates that portal messages from Black patients receive physician responses at lower rates than those from White patients, a structural disparity that may compound the trust deficit caregivers described [29]. Caregiver perspectives on AI-assisted interpretation of medical notes were mixed, reflecting both interest in tools that could bridge the clinical knowledge gap and concern that AI may be too general to address a specific child’s needs, a tension that warrants attention in future platform design. These findings extend prior work on documentation inconsistencies [15] by demonstrating that documentation problems directly affect caregiver trust and engagement. These documentation barriers may constrain caregivers’ ability to engage with and act on health information, a process prior research has linked to poorer asthma outcomes [10,17]. Rather than indicating individual disengagement, participants’ accounts point to EHR design as an underexamined factor shaping the gap between treatment availability and everyday asthma management. Addressing these barriers requires both technical solutions and a shift in how caregiver knowledge is recognized within health records [15].

Caregivers’ perspectives reflect knowledge emerging at the intersection of health and lived experiences, and their suggestions underscore that community-informed design is essential for addressing asthma disparities rooted in structural racism and environmental injustice, particularly for Black caregivers in urban settings like Philadelphia [6]. While the findings are specific to this population and context, they offer insights relevant to other marginalized caregiver communities managing pediatric chronic illness through health information systems designed primarily for clinical use. Improvements to EHR systems based on day-to-day experiences, including direct messaging with physicians, integration of environmental data such as pollen levels, the ability to search for symptoms with clinical follow-up, and improved medication refill processes, signify opportunities to enhance EHR design. The integration of their recommendations aligns with emerging frameworks for shared decision-making in asthma care [11] and broader efforts to develop patient-centered clinical decision support tools [16,30] to advance better outcomes.

Finally, caregivers described efforts to involve their children in managing their asthma care, including engaging them with their health records. However, existing systems often do not support age-appropriate engagement or youth-centered design [31]. These findings point to opportunities to develop interfaces and features that facilitate meaningful child participation and acknowledge young people’s distinct lived experiences with chronic illness.

### Implications for Developing a Community- and Family-Centered Medical Platform

Without structural changes to patient portal design, existing disparities in asthma management are likely to persist despite advances in clinical care. Platform design should integrate community knowledge alongside professional medical expertise rather than treat caregiver experience as supplementary. When caregivers access their patient portal, their documented observations and concerns should be visible alongside clinician notes, creating a shared record that positions caregiver knowledge as a legitimate input rather than a supplement [15].

Key platform features directly requested by participants include easy-to-understand translations of medical terminology, direct messaging with physicians, symptom-search functionality with clinical follow-up, and streamlined medication refill processes. The research team also proposes design directions informed by but extending beyond the data: AI-assisted interpretation of medical notes, integration of environmental data such as pollen levels, and caregiver-facing tools for annotating or correcting records. These represent researcher-generated extrapolations rather than direct caregiver recommendations, and they should be co-designed with community members before implementation [30]. Integrating environmental data, such as air quality and pollen counts, can help families identify and respond to environmental triggers [32]. Child-friendly interfaces could support developmental transitions toward independence by offering age-appropriate education and tools that help young people gradually assume self-management responsibilities.

Platforms should also facilitate peer-to-peer knowledge sharing through moderated community forums where caregivers exchange insights on managing asthma, identifying environmental triggers, and navigating care systems [33]. Customizable views can show only relevant information to different stakeholders while allowing caregivers to control what gets shared across the distributed network of parents, children, family members, and schools involved in asthma care [34].

Although this study centers on childhood asthma, these findings may extend to other pediatric chronic conditions in which caregivers carry substantial responsibility for coordination, monitoring, and interpretation outside clinical settings. More broadly, they suggest that patient-facing health information systems should treat caregiver knowledge as essential rather than peripheral.

### Limitations

This research has several noteworthy limitations. First, we interviewed only primary caregivers from one region in the United States; future research should include caregivers from diverse geographic locations to gain broader perspectives. Additionally, asthma diagnoses were caregiver-reported rather than clinically verified, and the distribution of intermittent versus persistent diagnoses was not systematically recorded, which limits the specificity of clinical context available for interpreting caregiver experiences. Second, all participants were Black female caregivers. Future studies should include fathers, grandparents, other guardians, caregivers from diverse racial and ethnic backgrounds, and children and adolescents with asthma themselves. Third, this qualitative study does not allow generalization to all caregivers of children with asthma. Because the recommended platform features have not yet been developed, caregiver suggestions reflect proposed rather than validated interventions. Future research should use participatory design methods to develop and evaluate these features with community members. Fourth, participants received a US $100 gift card incentive, which may have influenced willingness to participate and introduced self-selection bias; caregivers who chose to participate may differ systematically from those who did not in ways that shaped their portal experiences and perspectives.

### Implications for Future Research

Additional research is needed in four areas: (1) expanding participant diversity to include different caregiver types, children and adolescents with asthma, and multiple geographic locations and health care systems; (2) using participatory design to translate caregiver recommendations into platform features and testing whether these reduce caregiver burden, improve health outcomes, and advance health equity; (3) examining how health information systems intersect with environmental and health justice, and whether technology designed with community input reduces epistemic injustice by shifting power dynamics and enhancing caregiver agency; and (4) examining whether the community-centered, caregiver-as-expert model developed here is transferable to other pediatric chronic conditions in which caregivers carry primary responsibility for coordination, monitoring, and interpretation outside clinical settings.

### Conclusions

This study elicited Black primary caregivers’ perspectives on managing childhood asthma and their experiences with EHR-generated information and patient portals to inform the development of a community- and family-centered medical platform. In this study, participants described the profound emotional and practical work of asthma management, emphasized the importance of accessible health information and education, and articulated how family and community knowledge supports their confidence. When engaging with EHR-generated information and patient portals, the majority of primary caregivers used these systems selectively and strategically, identified barriers related to accuracy and medical terminology that undermined trust, and offered meaningful recommendations for improvement.

Our findings show that effective childhood asthma management requires more than clinical treatment. It requires information systems that honor caregiver expertise while reducing burden, provide transparent and accurate documentation, integrate environmental and social context, and actively work to reduce knowledge inequities. By centering caregivers’ voices through a partnership with a community organization, this research contributes to the design of platforms that advance health equity rather than perpetuate existing disparities.

## Supplementary material

10.2196/100502 Checklist 1 SRQR checklist.

## Abbreviations

EHR: electronic health record
SRQR: Standards for Reporting Qualitative Research
